# Usual Dietary Intake, Nutritional Adequacy and Food Sources of Calcium, Phosphorus, Magnesium and Vitamin D of Spanish Children Aged One to <10 Years. Findings from the EsNuPI Study †

**DOI:** 10.3390/nu12061787

**Published:** 2020-06-16

**Authors:** Esther Cuadrado-Soto, Ana M. López-Sobaler, Ana Isabel Jiménez-Ortega, Aránzazu Aparicio, Laura M. Bermejo, Ángela Hernández-Ruiz, Federico Lara Villoslada, Rosaura Leis, Emilio Martínez de Victoria, José Manuel Moreno, María Dolores Ruiz-López, María José Soto-Méndez, Teresa Valero, Gregorio Varela-Moreiras, Ángel Gil, Rosa M. Ortega

**Affiliations:** 1 Department of Nutrition and Food Science, Faculty of Pharmacy, Complutense University of Madrid, Plaza Ramón y Cajal S/N, 28040 Madrid, Spain; esther.cuadrado@ucm.es (E.C.-S.); araparic@ucm.es (A.A.); mlbermej@ucm.es (L.M.B.); rortega@ucm.es (R.M.O.); 2 UCM Research Group VALORNUT-920030, Complutense University of Madrid, Plaza Ramón y Cajal S/N, 28040 Madrid, Spain; aisabel.jimenezo@salud.madrid.org; 3 Pediatric Gastroenterology Unit, San Rafael Hospital, 28016 Madrid, Spain; 4 Iberoamerican Nutrition Foundation (FINUT), Av. Del Conocimiento 12, 3ªpta, Armilla, 18016 Granada, Spain; ahernandez@finut.org (Á.H.-R.); mdruiz@ugr.es (M.D.R.-L.); msoto@finut.org (M.J.S.-M.); agil@ugr.es (Á.G.); 5 Instituto de Nutrición Puleva, 18004 Granada, Spain; federico.lara@lactalis.es; 6 Department of Pediatrics, Unit of Pediatric Gastroenterology, Hepatology and Nutrition, University Clinical Hospital of Santiago, IDIS, University of Santiago de Compostela, 15706 Santiago de Compostela, Spain; mariarosaura.leis@usc.es; 7 CIBEROBN (Physiopathology of Obesity and Nutrition), Instituto de Salud Carlos III (ISCIII), 28029 Madrid, Spain; 8 Department of Physiology, Faculty of Pharmacy, University of Granada, Campus de Cartuja, s.n, 18071 Granada, Spain; emiliom@ugr.es; 9 Institute of Nutrition and Food Technology “José Mataix”, Biomedical Research Center, University of Granada, Parque Tecnológico de la Salud, Avenida del Conocimiento s/n, Armilla, 18100 Granada, Spain; 10 Pediatric Department, University of Navarra Clinic, Calle Marquesado de Sta. Marta, 1, 28027 Madrid, Spain; jmorenov@unav.es; 11 Department of Nutrition and Food Sciences, Faculty of Pharmacy, University of Granada, Campus de Cartuja, s.n, 18071 Granada, Spain; 12 Spanish Nutrition Foundation (FEN), c/General Álvarez de Castro 20, 1ªpta, 28010 Madrid, Spain; tvalero@fen.org.es (T.V.); gvarela@ceu.es (G.V.-M.); 13 Department of Pharmaceutical and Health Sciences, Faculty of Pharmacy, CEU San Pablo University, Urb. Montepríncipe, crta. Boadilla km. 5.3, Boadilla del Monte, 28668 Madrid, Spain; 14 Department of Biochemistry and Molecular Biology II University of Granada, University of Granada, Campus de Cartuja, s.n, 18071 Granada, Spain

**Keywords:** EsNuPI study, calcium, phosphorus, magnesium, vitamin D, pediatric nutrition, food sources, Spanish children, dairy products, infant formula

## Abstract

Bone problems in the population begin to be establish in childhood. The present study aims to assess the usual calcium, phosphorus, magnesium, and vitamin D intakes, along with the food sources of these nutrients, in Spanish children participating in the EsNuPI (Estudio Nutricional en Población Infantil Española) study. Two 24 h dietary recalls were applied to 1448 children (1 to <10 years) divided into two sub-samples: one reference sample (RS) of the general population [*n* = 707] and another sample which exclusively included children consuming enriched or fortified milks, here called “adapted milks” (AMS) [*n* = 741]. Estimation of the usual intake shows that nutrient intake increased with age for all nutrients except vitamin D. Using as reference the Dietary Reference Values from the European Food Safety Authority (EFSA), calcium and magnesium intakes were found to be below the average requirement (AR) and adequate intake (AI), respectively, in a considerable percentage of children. Furthermore, phosphorus exceeded the AI in 100% of individuals and vitamin D was lower than the AI in almost all children studied. The results were very similar when considering only plausible reporters. When analyzing the food sources of the nutrients studied, milk and dairy products contributed the most to calcium, phosphorus, magnesium, and vitamin D. Other sources of calcium were cereals and vegetables; for phosphorus: meat, meat products, and cereals; for magnesium: cereals and fruits; and, for vitamin D: fish and eggs. These results highlight the desirability of improving the intake concerning these nutrients, which are involved in bone and metabolic health in children. The AMS group appeared to contribute better to the adequacy of those nutrients than the RS group, but both still need further improvement. Of special interest are the results of vitamin D intakes, which were significantly higher in the AMS group (although still below the AI), independent of age.

## 1. Introduction

Osteoporosis is a major global public health concern, the prevalence of which increases with the life expectancy of the population [1]. Although it develops in advanced stages of life, it originates in the pediatric age during childhood and adolescence. Therefore maximizing bone mineral mass during these vital stages can decrease the risk of osteoporotic fractures in later life [2,3,4].

Throughout childhood, genetic factors determine ~70–80% of growth and acquisition of bone mineral content, while lifestyle determines ~20–30% [5]; among these exogenous factors, which can be modified, food deserves attention [6,7].

Calcium, phosphorus, magnesium, and vitamin D, which are the nutrients object of attention in this study, play important roles in the growth and development of the bone mass, which is at its maximum in the pediatric stage [2,8,9] regardless of having many human biological roles in body homeostasis, physiological, and cellular functions [10,11]. During childhood and adolescence an adequate intake of these nutrients can contribute to achieving an optimal peak of bone mass, which may help to prevent the development of osteoporosis in later stages of life [6].

However, the intake of many of the nutrients involved in bone remodeling (i.e., calcium, magnesium, and vitamin D) has been shown to be insufficient in a high percentage of children in developed populations [2,12,13,14,15]. Indeed, the United States Department of Agriculture (USDA) [1] has pointed calcium and vitamin D as insufficient in the diet and of public health concern; therefore, knowing their intake in children has become a priority.

Studies in children under the age of 10 in Spain are scarce and heterogeneous in methodology [12,13,16,17,18]. The “Alimentando la Salud del Mañana” (ALSALMA) study (carried out in 2013) assessed only the nutritional patterns of children under three years of age [16]. The National Dietary Survey on the Child and Adolescent Population project in Spain (ENALIA) study analyzed nutrient intake in children from 6 months to 17 years old, but was carried out from 2012 to mid-2014 [13]. The Anthropometry, Intake and Energy Balance Study (ANIBES) analyzed energy and nutrient intake, but was carried out only in children from 9 to 12 years old [12]. The Identification and prevention of Dietary- and lifestyle-induced health EFfectsIn Children and infantS (IDEFICS) study analyzed the intake of European children from 2 to 9 years old; however, this research did not include a representative sample of the Spanish population [18]. Furthermore, it is necessary to carry out studies constantly, in order to know the evolution of these issues and the most current situation of each population.

As dietary habits and dietary patterns begin to establish in early childhood and persist during adulthood [19], knowledge of overall nutrient intake—specifically, those involved in bone growth remodeling—give us an opportunity for intervention aimed at reducing the risk of suffering from various diseases and, in particular, osteoporosis and fragility [20,21].

On the other hand, considering that children have a high need for nutrients, their consumption of nutrient-rich foods is essential [22]. Specifically, regular consumption of dairy products and milk formulas can be useful in children’s health, as they are foods of high nutritional value which provide high amounts of macro- and micro-nutrients, (mainly calcium, magnesium, phosphorus, vitamin D and high quality protein) that are important for bone health [23,24]. In fact, some authors have found higher quality diets in children with higher dairy consumption, compared to those with lower consumption [14,18,25]. Nevertheless, it is interesting to analyze whether the consumption of adapted, enriched, or fortified milk formulas (generally called “adapted milks” in the present study) has potential benefits or disadvantages in the diets of children.

Therefore, the EsNuPI (“Nutritional Study in Spanish Pediatric Population”) study (performed in Spanish children aged one to <10 years old), aims to analyze the usual intake and dietary sources of calcium, phosphorus, magnesium, and vitamin D in children as well as analyzing the differences between those who usually consume adapted milks and those consuming standard milk, along with the factors associated with their intake.

## 2. Materials and Methods

### 2.1. Study Design and Sample

The data used in this work are part of the EsNuPI study, which is a prospective, cross-sectional, observational study, conducted from October 2018 to January 2019. All details about the design, protocol, and methodology of the EsNuPI study have been already described in detail elsewhere [26].

The EsNuPI study was planned to determine the eating habits, energy and nutrient intake, physical activities, and sedentary behaviors of Spanish aged one to <10 years, which were non-vegan and living in urban areas with >50,000 inhabitants. Two sub-samples were selected, one including children taking standard milk (a representative sample of Spanish children; RS) and one of convenience, which included children taking adapted, enriched, or fortified milks, which we called the “adapted milk consumers sample” (AMS). In addition, considering the need for representation of all age and sex groups, the sample was stratified (50% boys, 50% girls, 1 to <3 years old, 3 to <6 years old, and 6 to <10 years old) [26,27].

The EsNuPI study was conducted under the Helsinki declaration and was approved by the Ethics Committee of the University of Granada (No. 659/CEIH/2018) and registered in ClinicalTrials.gov (Unique Protocol ID: FF01/2019).

### 2.2. Procedures and Data Collection

The study information was collected in an initial face to face interview and in a second telephone interview at least 7 days later. The data collected are described below.

#### 2.2.1. Socio-Demographic and Anthropometric Information

In the first interview, a general questionnaire was applied to collect the following variables about children: place and date of birth, sex, academic level of parents or caregivers (elementary or less/secondary/university/higher education), place of residence, family income level, lifestyle, activity patterns, and sedentary behaviors of the child.

Height and weight data were declared by parents or caregivers, based on the child’s pediatric health card. Body mass index (BMI) was calculated and World Health Organization (WHO) reference standards were used to calculate BMI-Z scores [28,29]. BMI for age Z score was used to categorize children as ‘underweight’ (Z-BMI/age < −2 standard deviation (SD)), ‘normal BMI’ (Z-BMI/age −2 to +1 SD), or ‘overweight and obese’ (Z-BMI/age > +1 SD). Length/height for age Z score was used to categorize children as ‘stunting’ (Z-height/age < −2 SD), ‘normal height’ (Z-height/age −2 to +2 SD), or ‘high stature’ (Z-height/age > +2 SD).

#### 2.2.2. Physical Activity and Sedentary Behavior Questionnaire

To assess physical activity and sedentary behaviors, a modification of a questionnaire previously validated in children <10 years old from Colombia, based on the memory of activities carried out in a seven-day period, was applied [30].

The activities carried out by the child in one day (24 h) during the last week (a seven-day record) were recorded, including information on sleep hours and screen time; data for weekdays and weekend days were recorded separately. For more detailed information, see Madrigal et al. [26,27].

#### 2.2.3. Dietary Survey and Data Collection

Two 24 h dietary recalls (DR) were completed (one face-to-face and one by telephone) on non-consecutive days, including one weekday and one weekend day, using the parents or caregivers of the children to help determine the children’s food and drink consumption. Detailed information was requested on the dietary intake of the participants, indicating the ingredients, method of preparation, brands used for each dish consumed and the place of consumption (home or away). This information allowed us to code the products properly and to establish the weight consumed.

As supporting material for the correct completion of the dietary study, the interviewers used the “tables of common home measures, and habitual portion sizes for Spanish population” [31,32] and the “photo guide of common portions sizes of Spanish foods” [33], which was built using the “Pilot study for the Assessment of Nutrient intake and Food Consumption Among Kids in Europe” (PANCAKE) [34]. The photo guide included 12 food groups, 204 frequently consumed foods by Spanish children, and 944 photographs.

Furthermore, a software called “VD-FEN 2.1”, a Dietary Evaluation Program from the Spanish Nutrition Foundation (FEN) [33], was used to assess the reported intake of food, beverages, energy, and nutrients. This program uses Spanish food composition tables, with several expansions and updates [31].

In this paper, special attention was paid to the intake of nutrients most directly involved in bone remodeling to assess their reported intake and adequacy with respect to the European Food Safety Authority (EFSA) [35] recommendations. Furthermore, the calcium/phosphorus ratio was determined.

To determine the food sources of the nutrients under study, different food items were categorized into the following 18 food categories: “milk and dairy products”, “other dairy products”, “cereals”, “meat and meat products”, “oils and fats”, “bakery and pastry”, “fruits”, “vegetables”, “sugars and sweets”, “ready to cook/eat”, “beverages”, “legumes”, “eggs”, “fish and shellfish”, “appetizers”, “cereal-based baby foods and supplements”, “nuts”, and “sauces and condiments”. To clarify, “milk and dairy products” included yogurt, cheese, curd, and kefir; whereas “other dairy products” included dairy desserts, shakes, ice cream, cream, and condensed milk.

An analysis of the total amount of calcium, phosphorus, magnesium, and vitamin D provided by all the foods eaten by the population was performed. The population proportion method [36] was used to determine the contribution of each nutrient from the different food categories. Specifically, the calcium content specified in each food and beverage was added, according to its intake by everyone in the group. This calcium was compared to the total calcium consumed, which was obtained from the sum of the total calcium ingested by all participants during the two days. The percentage of calcium from each group was calculated as follows: (sum of calcium from food group (mg)/total sum of calcium from all foods (mg)) × 100 [36]. The same procedure was performed for the rest of nutrients studied (i.e., phosphorus, magnesium, and vitamin D).

### 2.3. Evaluation of Plausible Under- and Over-Reporters (Misreporting)

Intentional (as well as unintentional) misreporting, which comprises under- and over-reporting, are well-known problems in dietary assessments, which should be evaluated when conducting a dietary study [37]. Misreporting of energy intake for the EsNuPI study has been previously reported [27].

Subjects were identified as plausible under- or over-reporters of energy intake, considering the relationship between their energy intake (EI) and Basal metabolic rate (BMR), which was estimated using the equation of Schofield [38]. Given these criteria, under-reporters were identified as those with EI/BMR ratios up to 0.97–1.00, while over-reporters were identified by EI/BMR ratios above 2.45–2.58, depending on the subject’s age and sex.

It is important to assess misreporting; however, following the EFSA [39] recommendations, it is not appropriate to exclude potential misreports from the study. This is because the exclusion of misreports from the database can introduce bias into the research results. Therefore, they must be identified, but not excluded from the database.

### 2.4. Statistical Analysis

Once the dietary information was collected, the 746 foods reported by the children were grouped into 18 food groups and transformed into energy and nutrients for the analysis of the results.

As the average intake obtained by applying a 24 h dietary recall for only a small number of days (observed intakes) does not adequately represent the usual intake, it is necessary to apply a statistical model to eliminate the day-to-day variation in food consumption [40]. To this end, the method developed by Nusser et al. [41]—also known as the Iowa State University (ISU) method—was applied. This method consists of three steps: a transformation step, mapping the intake to a normal scale; an estimate of the usual intake by using a measurement error frame; and, finally, a retro-transformation step, to return the estimated usual intakes to their original scale.

The ISU method was implemented using the PC-SIDE software (version 1.0, 2003) (Iowa State University, Ames, IA, USA), which was designed for this purpose. This program estimates the percentiles of usual nutrient intake distributions and the percentages that remain above or below the dietary reference cut-off values. Whether the intake corresponded to the first 24 h DR performed in the initial interview or to the telephone interview was considered in the adjustment of dietary data, stratifying by sex, age group, and group membership (i.e., RS or AMS).

To assess nutrient adequacy, we used the Dietary Reference Values from EFSA [35], which includes reference values for calcium, phosphorus, magnesium, and vitamin D. The proportion of the population with usual intakes less than the average requirement (AR) provides an estimate for the proportion of the group whose intakes did not meet the nutrient requirements. For children aged 1 to <10 years, there are only established ARs for calcium, whereas for phosphorus, magnesium, and vitamin D, there are established adequate intakes (AIs). These AIs can be used to determine the proportion of individuals with adequate nutrient intake.

To describe the dietary intakes of the participants by sample group (RS and AMS), and by sex and age groups, the mean, standard deviation (SD), median, and 5th and 95th percentiles were used for continuous variables, and frequencies and percentages for categorical variables. For this analysis, the population was divided into those groups established by EFSA, according to age (calcium, phosphorus and vitamin D: 1 to <4 years, 4 to <6 years, and 6 to <10 years; magnesium: 1 to <3 years, 3 to <6 years, and 6 to <10 years) [35].

The Kolmogorov–Smirnoff normality test was used to check the normality of the distribution of the variables to decide between parametric or non-parametric analyses for comparisons. For variables that did not follow normality, appropriate non-parametric statistical tests were used for group comparisons.

Mann–Whitney U and Chi-squared tests were used to evaluate differences between the reference and adapted milk consumer samples (in the total sample and by age group). Variance analysis (ANOVA) with Bonferroni correction was used to make multiple comparisons or Kruskal–Wallis analysis to calculate differences between each age group within the established groups. Student’s *t*-test or Chi-squared test was used to evaluate adequacy differences between samples (RS and AMS) by sex and age groups. Kruskal–Wallis or Z-test with Bonferroni correction were used to perform multiple comparisons by age group between samples. Linear correlation and logistic regression analyses were also applied, in order to ascertain the influence of various variables on the intakes of the nutrients under study.

The level of significance was set at *p* < 0.05. Statistical analyses were performed using the statistical software package SPSS version 24.0 for Mac OS (IBM Corp., Armonk, NY, USA).

## 3. Results

### 3.1. Description of the Sample

The final EsNuPI sample included 1448 children (49.7% girls and 50.3% boys) between the ages of 1 and <10 years, whose parents or caregivers agreed to completed two 24 h DR. The RS group represented 48.8% of the sample studied and the AMS represented 51.2%.

Anthropometric, sociodemographic, and activity data are presented in Table 1, as well as data on consumption of supplements and number of feeding bottles or glasses of milk consumed per day.

In AMS, there was a higher percentage of children aged 1 to <6 years and, in RS, there were more children aged 6 years and older. This could explain why the weight and height of the AMS children were lower than those observed in RS (Table 1).

The consumption of dietary supplements very low, only 0.4% of RS and 0.7% of AMS took vitamin D supplements and 0.7% of RS and 0.7% of AMS took a polyvitamin with minerals or vitamin complexes (Table 1).

### 3.2. Usual Calcium, Phosphorus, Magnesium, and Vitamin D Intake in Children under Study

Table 2 presents the usual daily intake of calcium, phosphorus, magnesium, and vitamin D in the whole population and separately by age and sex groups, as well as differentiating between the RS and the AMS. Different age ranges were used for calcium, phosphorus, and vitamin D, as compared to Magnesium, according to the RI/AI marked by EFSA [35].

There were no sex differences in the intake of the nutrients studied, except for magnesium, for which boys (194 ± 45 mg/day) had a significantly higher intake than girls (188 ± 41 mg/day).

The intake of calcium (r = 0.283), phosphorus (r = 0.570), and magnesium (r = 0.316) increased with age (*p* < 0.001). However, vitamin D intake (r = −0.191) decreased with age (*p* < 0.001; Table 2).

#### 3.2.1. Calcium

Calcium intake was lower than AR in a small percentage of children aged 1–3 years, but the percentage increased in children aged 6–10 and especially in those aged 4–6. As a result, the percentage of children aged 4 and over with calcium intake below AR was 24.5% in RS boys (compared to 26.7% in RS girls) and 8.1% in AMS boys (compared to 17.5% in AMS girls). It should be emphasized that the calcium intake was more adequate (significantly higher) in children aged 6 and over in the AMS group, compared to those in the RS group (Table 2).

Having a higher level of education in one parent or being underweight were factors in preventing calcium intake from being similar to or higher than the P50, in the RS group (Table 3), while taking two or more feeding bottles or glasses of milk per day was a factor that helped to achieve a calcium intake above the median both in the RS and AMS groups (Table 3 and Table 4).

Usual calcium intake showed a weak positive (but significant) association with the height z-score in the total sample (r = 0.126; *p* = 0.000). By age, this positive correlation was found in children aged 4 to 5 years (r = 0.105; *p* = 0.018) and those 6 years and older (r = 0.134; *p* = 0.003).

#### 3.2.2. Phosphorus

The intake of phosphorus in all cases exceeded AI, and was significantly lower in 1–3 years children of the AMS group, compared to those of the RS group (Table 2).

Having secondary education as highest level of education in one parent was associated with lower phosphorus intake in RS children. Those living in areas with larger populations (>300,000 people) were more likely to have intakes of phosphorus above the median (AMS), while having a delay in the height for age (or stunting) in AMS group was associated with a greater difficulty in achieving phosphorus intake similar to or greater than median (Table 3 and Table 4).

In the EsNuPI study, the ratio for calcium to phosphorus for the whole population was 0.78 ± 0.16 (RS: 0.74 ± 0.13 vs. AMS: 0.82 ± 0.16), which was very low compared to the recommendations. Specifically, 93.6% of the studied children had a calcium/phosphorus ratio below 1/1. This relationship decreased with age, but was significantly higher in the AMS group than in RS in all age categories considered (RS: 0.84 ± 0.18 vs. AMS: 0.90 ± 0.21 for children aged 1–3 years; RS: 0.72 ± 0.1 vs. AMS: 0.78 ± 0.11 in children aged 4–5 years; and RS: 0.69 ± 0.09 vs. AMS: 0.76 ± 0.09 for children aged 6 years and older).

#### 3.2.3. Magnesium

Average and median magnesium intakes were close to the AI, although the percentage of children aged 3 to <6 years that exceeded the AI was low. Children aged 3 to <6 years and 6 to <10 years in the AMS group had significantly lower magnesium intakes than those in the RS group (Table 2).

Larger population size (>300,000 people) or undeclared income (Not known/no answer) were associated to have magnesium intake similar to or greater than median in AMS children (Table 4). Additionally, having higher incomes (>2000 €/month) was associated with higher magnesium intake in RS children (Table 3).

#### 3.2.4. Vitamin D

The intake of vitamin D was significantly higher in AMS boys and girls, in all age groups, compared to RS children (Table 2). Only 0–0.6% of children in RS and 0.4–4.3% of children in AMS exceeded the AI.

Taking two or more feeding bottles or glasses of milk per day was a factor that helped to achieve vitamin D intake similar to or greater than median in AMS children (Table 4). Furthermore, those living in higher population areas (>300,000 people) were more likely to have intakes of vitamin D >P50 in RS children, while the same was associated with lower vitamin intake in AMS children.

#### 3.2.5. Calcium, Phosphorus, Magnesium and Vitamin D in Plausible Reporters

The percentage of plausible energy reporters was high for both groups (84.7% in the RS group and 83.5% in the AMS group) [27]. Therefore, the data presented in the rest of this report have not been adjusted for misreporting.

Nevertheless, considering data of the usual daily intake of calcium, phosphorus, magnesium, and vitamin D of the plausible reporters only (Supplementary Table S1), very similar results were obtained to those found in the total sample.

Calcium intake was significantly higher in boys aged 6 and over in the AMS group, compared to those in the RS group; however, in plausible reporters, the difference was not significant in girls. The intake of phosphorus was significantly higher in males aged 1–3 years of the RS group, compared to those in the AMS group, and in boys and girls aged 4 to <6 years in the RS group, compared to the AMS group.

Regarding the calcium–phosphorus ratio, magnesium, and vitamin D, the situation was very similar to that obtained for the total sample.

### 3.3. Contribution of Food Sources to Calcium, Phosphorus, Magnesium and Vitamin D Reported Intakes

**Figure 1:**
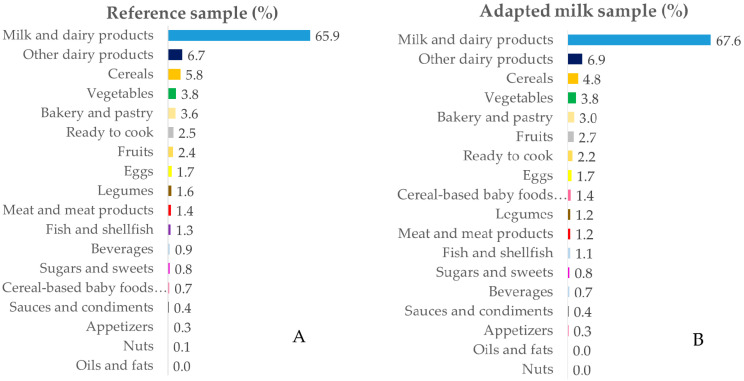
Percentage of the 18 food groups sources, in terms of the total calcium intake (%), among the reference sample (**A**) and the adapted milk consumer sample (**B**) of the Spanish Pediatric Population (EsNuPI) study.

**Figure 2:**
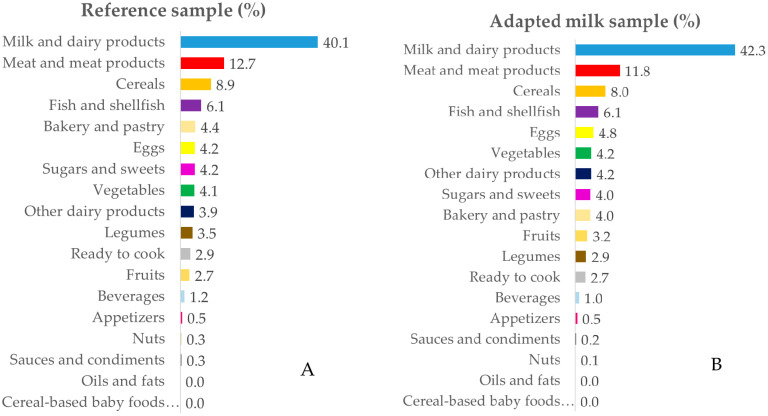
Percentage of the 18 food groups sources, in terms of the total phosphorus intake (%), among the reference sample (**A**) and the adapted milk consumer sample (**B**) of the Spanish Pediatric Population (EsNuPI) study.

**Figure 3:**
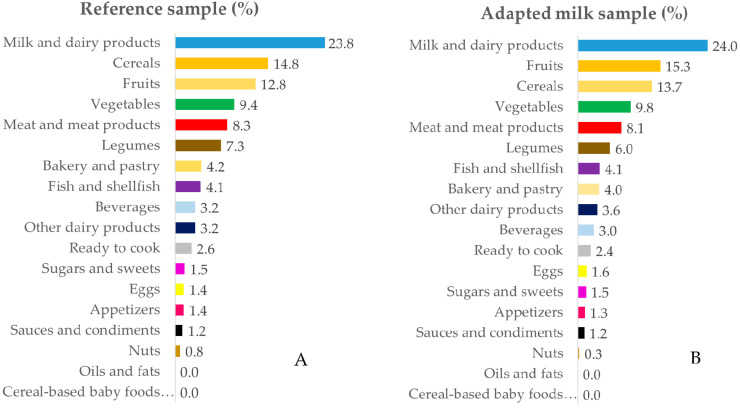
Percentage of the 18 food groups sources, in terms of the total magnesium intake (%), among the reference sample (**A**) and the adapted milk consumer sample (**B**) of the Spanish Pediatric Population (EsNuPI) study.

**Figure 4:**
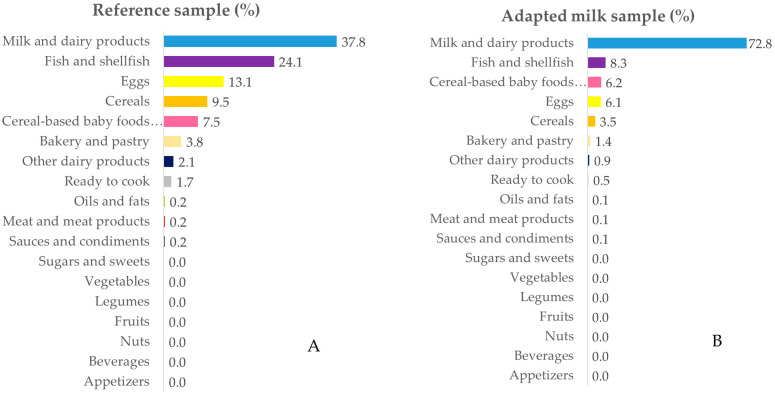
Percentage of the 18 food groups sources, in terms of the total vitamin D intake (%), among the reference sample (**A**) and the adapted milk consumer sample (**B**) of the Spanish Pediatric Population (EsNuPI) study.

The intake data were grouped into 18 food groups for in-depth analysis. Figure 1, Figure 2, Figure 3 and Figure 4 represent the contribution (%) of food and beverage categories to the daily calcium, phosphorus, magnesium, and vitamin D reported intakes for the whole population. The data obtained for children in RS and AMS are summarized, and the food groups are presented in decreasing order of the amount of the nutrients they contribute to the daily intake.

#### 3.3.1. Calcium

The main sources of calcium for RS and AMS children were milk and dairy products, followed by other dairy products, cereals, vegetables, bakery and pastry, ready to cook/eat, fruits, and eggs. It can be noted that, in AMS children, cereal-based baby foods and supplements contributed 1.4% of the calcium; whereas, in RS children, they contributed 0.7% (Figure 1A,B). No major differences were observed when considering each age block separately (Supplementary Table S2).

#### 3.3.2. Phosphorus

The largest source of phosphorus was milk and dairy products, followed (in both groups) by meat and meat products, cereals, fish, and shellfish. Foods that provided smaller amounts of phosphorus included bakery and pastry, eggs, sugars and sweets, and vegetables (Figure 2A,B). The above decreasing contribution order was maintained when considering different age groups (Supplementary Table S3).

#### 3.3.3. Magnesium

Milk and dairy products were the main sources of magnesium for both RS and AMS groups, followed by cereals and fruits. Other relevant sources of magnesium were vegetables, meat and meat products, and legumes. The rest of the sources contributed less than 4.2% of the magnesium ingested (Figure 3A,B). The specific data characteristics of each age group are specified in Supplementary Table S4.

#### 3.3.4. Vitamin D

The main source of vitamin D was milk and dairy products in both groups, followed by fish, eggs, cereals, cereal-based baby foods, supplements, and bakery and pastry (Figure 4A,B; see Supplementary Table S5 also).

## 4. Discussion

This paper provides recent estimates of the calcium, phosphorus, magnesium, and vitamin D intake, together with the food sources of these nutrients, in a representative sample of Spanish infants, toddlers, and children (1 to <10 years), as well as analyzing the differences between those who usually take adapted and fortified milk formulas versus standard milk. Furthermore, the influences of different factors (e.g., sociodemographic, anthropometric, parental educational level, and so on) on the intake of some of these nutrients were also analyzed.

The anthropometric, sociodemographic, and physical activity data of the children studied were similar to those found in other studies of Spanish children of a similar age [13,14,20,42,43,44].

Among the nutrients studied, calcium and vitamin D have been the most frequently analyzed, in relation to bone mass [4,12,14,20,45,46,47]. However, it is also important to pay attention to magnesium in relation to children’s bone health. Abrams et al. [9] in a sample of 63 healthy children aged 4 to 8 years found that magnesium intake but not calcium intake, was significantly associated with both total bone mineral content and density.

### 4.1. Calcium

Calcium intake in RS and AMS groups was similar to that found in Spain by Olza et al. [12], in children in the ANIBES study aged 9–12 years (872 ± 22 mg/day in boys and 759 ± 26 mg/day in girls), and also to those found by Dalmau et al. [20], in children in the ALSALMA study aged 1–2 years (795.1 mg/day) and 2–3 years (858.6 mg/day); by Ortega et al. [14], in children aged 7–11 years (859.9 ± 249.2 mg/day); by Chouraqui et al. [45] (770–792 mg/day in children of 12–23 months and 729–746 mg/day in children of 24–35 months) and by Jiménez-Aguilar et al. [46] (759.3 mg/day in children of 1–2 years and 859.9 mg/day in children of 3–4 years). However, it was slightly lower than that found in the ENALIA study [13] (928 ± 178.5 and 879 ± 161.9 mg/day in boys and girls of 1–3 years; 956 ± 159.1 and 903 ± 147.6 in boys and girls of 4–8 years; and 1025 ± 212.5 and 959 ± 167.7 mg/day in boys and girls of 9–13 years, respectively) and in the “Ingesta de calcio y densidad mineral ósea en escolares españoles” (CADO) study in children of 5–12 years (1227 ± 404 mg/day in boys and 1163 ± 401 mg/day in girls) [4]; it was also slightly lower than that found by other authors in other countries [19,22,48].

A review examining nutrient intake in nine European countries (i.e., Belgium, Denmark, France, Germany, the Netherlands, Poland, Serbia, Spain, and the UK) indicated that the intake of calcium in children aged 4–10 years ranges from 563 mg/day to 1106 mg/day [47], so the intake found in this paper was within the range observed by these authors.

Calcium intake increased with age, as has been found in other studies [13,46]; but as the AR also increases with age, the percentage of children with <AR intakes was higher in the older groups, especially in those of 4 to <6 years (Table 2 and Supplementary Table S1).

The percentage of inadequate calcium intake was similar to that observed in other studies in children of similar ages [20,22,45,49]. Calcium intake was significantly higher in children aged 6 and over in the AMS group, compared to those in the RS group (Table 2). Even though there is still controversy, several studies have analyzed the convenience of using products adapted to the higher nutrient needs of children [22,45]. Most of these studies have been conducted in young children (aged 1–3 years), in which authors such as Chouraqui et al. [45] have pointed out that the consumption of young-child formulas may help infants and children at risk of nutrient deficiencies to meet their nutritional requirements. These authors found that, at all ages studied, the children consuming young-child formula had higher intakes of several nutrients, especially vitamin D; although they also mentioned that vitamin D intake remained below the EFSA AI. Eussen et al. [22] also indicated that the replacement of habitual cow’s milk intake (in children 12–18 months) by a matching volume of 300 mL of young-child formula may lead to nutritional intakes close to recommendations in young children. The European Society for Pediatric Gastroenterology, Hepatology, and Nutrition (ESPGHAN) Committee on Nutrition has suggested that, based on available evidence, there is no necessity for the routine use of young-child formula in children from 1 to 3 years of life, but that they can be used as part of a strategy to increase the intake of iron, vitamin D, and n-3 PUFA and decrease the intake of protein, compared with unfortified cow’s milk [50]. Coinciding with the benefit observed by Chouraqui et al. [45] associated with the consumption of young-child formula, a higher calcium intake was found in children aged 6–10 years taking AMS in this research, showing that membership to the AMS group was associated with a higher likelihood of having higher calcium intake than median.

Some authors [51] have suggested that socioeconomic status is a predictor of higher calcium and vitamin D intake, and that physical activity was also correlated to daily calcium and vitamin D intake; however, this type of influence was not observed in our study. However, higher income was a positive factor in achieving phosphorus and magnesium ≥P50 intakes. Considering the highest level of education achieved by one of the parents, it was observed that university education in one parent was a factor in preventing calcium intake from being similar to or higher than the P50 (Table 4). These results coincide with those of Tornaritis et al. [52], who also found no influence on the intake of these nutrients in children from 6 to 18 years of age, depending on the educational level of their mother.

We found a weak positive correlation between usual calcium intake and height z-score. This result was also observed by Rubio-López et al. [49].

### 4.2. Phosphorus

The results obtained for phosphorus intake were similar to those observed in other studies [12,20,22,45,48] and slightly lower than those obtained in other research [13,19] for children of a similar age (Table 2 and Supplementary Table S1).

Intake of phosphorus exceeded AI in 100% of the children studied and, as in other investigations [12,13], its contribution can be considered as sufficient and, in some age groups, even excessive [53].

### 4.3. Calcium–Phosphorus Ratio

Authors such as Loughrill et al. [54] have suggested that adequate intake of calcium and phosphorus should be in the appropriate ratio of 1–2:1. Poor calcium absorption can be magnified if the calcium–phosphorus ratio is inadequate [14,49,55,56,57].

As calcium intake was lower than AR in a variable percentage of children and phosphorus intake was higher than AI, this ratio was frequently lower than the recommended ratio, as has been found in other studies [14,49,55]. Specifically, 93.6% of the studied children had a calcium–phosphorus ratio below 1. Rubio-López et al. [49] also found that 99% of studied children had a calcium–phosphorus ratio below 1.

However, the calcium–phosphorus ratio was significantly higher in AMS than RS, a difference that remained in all age groups. This is a benefit for the AMS group, which has a higher calcium intake and higher calcium/phosphorus ratios in their diets; given that low calcium intake associated with high phosphorus intake may have an adverse effect on the use of calcium and maintenance of bone mass in children [49,54,55].

### 4.4. Magnesium

The magnesium intake found in the EsNuPI study was similar to that found by other authors [12,19,20,45], and somewhat inferior to that found by López-Sobaler et al. [13].

Taking into account the review of nutrient intake in nine European countries by Mensink et al. [47], for magnesium and considering children aged 1–3 years, the lowest mean intake was observed in the U.K. (154 mg/day) and the highest in Belgium (191 mg/day). For children aged 4–10 years, mean intakes ranged from 185 mg/day for girls in the U.K. to 290 mg/day for German boys [47]; therefore, the intake found in the EsNuPI study would be within the extremes mentioned by these authors.

The percentage of children aged 6 to <10 years who exceeded the AI was low. This situation was similar (although somewhat better) to that mentioned by Tornaritis et al. [52], who found a high prevalence of inadequacy in magnesium intake (85.0–89.9%) in children aged 9–13.9 years.

Higher income (i.e., a family income of >2000 €/month) was associated with higher magnesium intake, which coincides with studies which have indicated that children with a lower socioeconomic level have lower magnesium intakes [58,59].

### 4.5. Vitamin D

Intake of vitamin D was similar to or higher than that found in other studies [4,12,13,20,22,60], especially when we consider the AMS children. Chouraqui et al. [45] indicated that the intake was much higher in children (12–35 months) consuming young-child formula than in those not consuming such products. Similarly, in the present study, vitamin D intake was significantly higher in AMS children than in RS (Table 2), and membership to the AMS group was associated with a greater possibility of having a vitamin D intake that exceeded the median intake for this vitamin.

The intake of vitamin D in the present study, especially considering children in the AMS group, was higher than that found by Mensink et al. [47], who found intakes ranging from 1.3 µg/day (for Poland) to 2.3 µg/day (for Belgium) in children aged 1–3 years. For girls aged 4–10 years, mean intake ranged from 1.6 µg/day (for Spain and Germany) to 2.9 µg/day (for The Netherlands; age 7–10 years). For boys aged 4–10 years, mean intake ranged from 1.9 µg/day (for Spain, France, Germany and the UK) to 3.5 µg/day (for the Netherlands; age 7–10 years).

As indicated by other authors [4,12,13,20,22,47,61,62], the situation regarding vitamin D is very worrying, as almost all children did not achieve AI (Table 2). Vitamin D plays an important role in bone health and has been related to many other sanitary and functional benefits; thus improving its intake is a priority [63].

Whereas it is difficult to achieve AI for vitamin D, the use of fortified foods (i.e., dairy and cereals) may be helpful as, although vitamin D is synthesized in the skin by the action of ultraviolet light, data from across the world indicate that hypovitaminosis D is widespread and is considered a public health problem. Deficiency of vitamin D has been reported in some selected Spanish populations, due to insufficient intake, sedentary lifestyles with low sun exposure, and use of sunscreen lotions [47,64].

### 4.6. Nutrient Sources

In order to improve the situation of children, in relation to calcium, phosphorus, magnesium, and vitamin D intake, it is useful to know the food source of the nutrients under study.

Regarding the sources of nutrients in other studies, it should be noted that these differ from one study to another, reflecting the differences in the usual dietary patterns as well as the different fortification policies of each country. In addition, it should be noted that the classification of foods may be different in each study. All of these factors make it difficult to compare data from the different studies.

However, both in EsNuPI and in other studies, milk and dairy products were the main source of all the nutrients studied, especially for calcium [12,14,24,42,61,65,66,67,68]. In fact, it was found that drinking two or more feeding bottles or glasses of milk a day is a factor that helps to achieve calcium and vitamin D intake above the median. However, despite being the main source of various nutrients, the consumption of milk and dairy products has decreased in children in recent decades and in many countries, contributing to many children not complying with the AR/AI of various nutrients [24]. In the EsNuPI Study, the milk consumed in the AMS group includes fortified and adapted formulas whose composition is adapted, being usually enriched with vitamin D in contrast to standard milk. This was reflected in the intakes of vitamin D in AMS children that were higher than those in RS children, although the intakes remained below the AI in most children. Similarly, Chouraqui et al. [45] noted that the consumption of young-children formulas may help infants and children (1–3 years) which are at risk of nutrient deficiencies, in order to meet their nutritional requirements. Furthermore, Huybrechts et al. [65] concluded that the consumption of fortified (growth) milk in children could be recommended to increase children’s vitamin D intake.

As a source of calcium, dairy products were followed by other dairy products (including dairy desserts, flavored milk, ice cream, cream, and condensed milk). It is difficult to compare these results with those of other studies, as the foods are not always grouped the same. For example, sweetened milk drinks were the second dietary source of calcium in Flemish preschoolers [65], contributing 22.8% to calcium intake—almost as much as milk. According to ESPGHAN recommendations [69], the intake of free sugars should be reduced and minimized, with a desirable goal of <5% energy intake in children and adolescents aged from 2–18 years, and their intake should probably be even lower in infants and toddlers under 2 years.

In addition, cereals are an important source of magnesium and vitamin D. The importance of consumption of grains and enriched breakfast cereals with various nutrients has been associated with increased magnesium intake in children [70], and analyzing the average American diet, Hess et al. (2019) pointed out that grains were the least expensive source of magnesium [57]. In addition, fortified breakfast cereals are one of the main sources of vitamin D and other nutrients [61].

Taking these data into account, it is desirable to approximate the consumption of dairy products and cereals to the recommended consumption.

Finally, it is noteworthy that, in children who did not take adapted formulas (RS), fish and eggs were good sources of vitamin D. Both fish [65] and eggs [71] have been found to be good sources of vitamin D in children. Although these foods are the first source of vitamin D in the present study, it is possible that children may not be meeting the recommended amounts of these foods. Therefore, adequate consumption of these two food groups should be considered and improved as early as childhood. In addition, the consumption of enriched foods or beverages with vitamin D could improve the intake of this vitamin. In AMS children, the intake of vitamin D was higher than in the RS, due to the contribution of fortified dairy products; however, their intake situation has room for improvement.

### 4.7. Strengths and Limitations

One of the main strengths of this study is being representative of the sample of children aged 1 to <10 years, as this is a group that has been little studied. Furthermore, it provides very recent information and analyzes the differences between the intakes of the nutrients studied between children in the reference sample and in the adapted milk consumers’ sample. Although dietary surveys are prone to underestimating dietary intake, which influences the estimation of the percentage of inadequate nutrient intakes, we have analyzed the degree of misreporting of the population in our study, analyzing separately the data from those children with plausible reports. On the other hand, the use of only two dietary recalls does not adequately reflect an individual’s usual intake. In our study, the observed nutrient intakes were transformed into usual intakes using the Nusser method [41], which allowed for a better estimation of the distribution of nutrient intakes and of the percentage of intakes lower than AR.

As for the limitations of the EsNuPI study, it can be noted that it had a cross-sectional design, which allows to know the situation at that time without establishing a causal relationship between nutrient intake with the bone health of children; therefore, the deepening of this topic should be the subject of future research. Additionally, it analyzed only populations from urban areas, not considering rural areas.

## 5. Conclusions

The usual nutrient intakes in the EsNuPI study met or exceeded their requirements, except for those of vitamin D. It is worth considering the percentage of children who did not achieve AR for calcium and AI for magnesium, from which it can be said that the situation in nutrients involved in bone health and other health aspects is clearly improvable, with the situation in AMS children being more favorable than in RS; although the situation in AMS children should also still be improved, as it is far from optimal.

When analyzing the sources of calcium, magnesium, phosphorus, and vitamin D, milk and dairy products were the first sources, in all cases, in this representative sample of Spanish children from 1 to <10 years. However, despite being the main source of various nutrients, the consumption of milk and dairy products has decreased in children in recent decades and in many countries, contributing to many children not complying with the AR/AI of various nutrients [24]. Based on the present study, adapted milk could be an effective strategy for overcoming the deficiencies of micronutrients (especially vitamin D in children) which are involved in important biological roles in the body, especially in bone health [4,12,14,20,45,46,47,72].

Considering that the calcium and magnesium intakes were below the AR/AI in a significant percentage of children, that phosphorus exceeded the AI in 100% of individuals, and that vitamin D was found, in practically all those studied, to be below the AI, it seems appropriate to improve the situation of these nutrients, which are involved in bone health. The AMS group appears to be in somewhat better shape than the RS, but still needs further improvement. Of special interest are the results of vitamin D intakes: although still below the AI, they were significantly higher in the AMS group, independent of the age of children.

## Figures and Tables

**Table 1:** General, anthropometric, and socioeconomic data by sex and age group in the Estudio Nutricional en Población Infantil Española (EsNuPI) population. Differences between reference sample and adapted milk consumer sample (*n* = 1448).

	Reference Sample			Adapted Milk Consumer Sample		
	Total	Boys	Girls	Total	Boys	Girls
n	707	357	350	741	371	370
Age group, n (%)						
1 to <3 years	162 (22.9) *	84 (23.5) *	78 (22.3) *	294 (39.7) *	144 (38.8) *	150 (40.5) *
3 to <6 years	244 (34.5) *	122 (34.2) *	122 (34.9) *	262 (35.4) *	128 (34.5) *	134 (36.2) *
6 to <10 years	301 (42.6) *	151 (42.3) *	150 (42.9) *	185 (25) *	99 (26.7) *	86 (23.2) *
Anthropometric characteristics, X ± SD						
Weight (kg) ^#^	20.8 ± 8.4 *	21.2 ± 8.5 *	20.5 ± 8.2	17.4 ± 7.4 *	17.9 ± 7.8 *	16.9 ± 6.9
Height (cm) ^#^	109.4 ± 20.1 *	110.1 ± 20.2 *	108.6 ± 20.0	100.5 ± 19.3 *	101.7 ± 20.2 *	99.2 ± 18.4
BMI (kg/m^2^) ^#^	16.9 ± 2.9	16.9 ± 3.1	16.8 ± 2.7	16.8 ± 2.9	16.8 ± 2.6	16.8 ± 3.1
Z-BMI/Age ^#^	0.59 ± 1.73	0.64 ± 1.97	0.54 ± 1.44	0.57 ± 1.72	0.52 ± 1.67	0.61 ± 1.77
Z-Weight/Height ^#^	0.59 ± 1.79	0.61 ± 2.09	0.57 ± 1.42	0.62 ± 1.69	0.53 ± 1.61	0.71 ± 1.76
Z-Height/Age ^#^	−0.25 ± 1.7 *	−0.14 ± 1.82 *	−0.36 ± 1.57	−0.54 ± 1.82 *	−0.52 ± 1.85 *	−0.55 ± 1.79
Physical activity (PAL) (X ± SD)						
1 to <3 years	1.56 ± 0.32	1.60 ± 0.32	1.53 ± 0.31	1.54 ± 0.30 a	1.54 ± 0.29 a,b	1.53 ± 0.30
3 to <6 years	1.56 ± 0.22	1.59 ± 0.21	1.52 ± 0.23	1.52 ± 0.22 a	1.53 ± 0.24 a	1.52 ± 0.19
6 to <10 years	1.58 ± 0.21	1.59 ± 0.22	1.57 ± 0.20	1.61 ± 0.21 b	1.62 ± 0.21 b	1.60 ± 0.21
Size of the municipality n (%)						
50,001–300,000 people	376 (53.2)	193 (54.1)	183 (52.3)	406 (54.8)	204 (55.0)	202 (54.6)
>300,000 people	331 (46.8)	164 (45.9)	167 (47.7)	335 (45.2)	167 (45.0)	168 (45.4)
Highest level of education achieved by one of the parents, n (%) ^¥^						
≤10 years of education	22 (3.2)	9 (2.6)	13 (3.8)	16 (2.2)	8 (2.2)	8 (2.2)
Secondary education	428 (62.3)	227 (65.4)	201 (59.1)	420 (57.9)	210 (58.0)	210 (57.7)
University studies	237 (34.5)	111 (32.0)	126 (37.1)	290 (39.9)	144 (39.8)	146 (40.1)
Family income, n (%)						
≤2000 €/month	297 (42.0)	146 (40.9)	151 (43.1)	297 (40.1)	148 (39.9)	149 (40.3)
>2000 €/month	226 (32.0)	123 (34.5)	103 (29.4)	238 (32.1)	110 (29.6)	128 (34.6)
Not known/no answer	184 (26.0)	88 (24.6)	96 (27.4)	206 (27.8)	113 (30.5)	93 (25.1)
Dietary supplements, n (%)						
Vitamin D, n (%)	3 (0.4)	3 (0.8)	0 (0.0)	5 (0.7)	4 (1.1)	1 (0.3)
Multivitamins and Minerals or Vitamins, n (%)	5 (0.7)	2 (0.6)	3 (0.9)	5 (0.7)	3 (0.8)	2 (0.5)
Number of feeding bottles or glasses of milk per day, n (%)						
Less than 2	222 (32.9)	110 (32.0)	115 (33.8)	178 (24.1)	92 (24.9)	86 (23.3)
2 or more	459 (67.1)	234 (68.0)	225 (66.2)	561 (75.9)	278 (75.1)	283 (76.7)

^#^ Variable that does not follow a normal distribution. ^¥^ Only information on 1413 children is available. SD: standard deviation; BMI: body mass index. Z-BMI/age: z-score for BMI for age. PAL: physical activity level. The PAL was calculated for individual and group level according to the European Food Safety Authority (EFSA) protocol, in order to assess misreporting (EFSA, 2013). * Significant differences between the reference sample and adapted milk consumer sample (in the total sample and by sex) are shown, applying the Chi-square and Mann–Whitney tests. Different superscript letters (a,b) indicate differences between age groups in the same column (same sex and same sample type: reference sample (RS) or adapted milks (AMS)), applying ANOVA tests. A *p*-Value < 0.05 was considered statistically significant.

**Table 2:** Daily calcium, phosphorus, magnesium, and vitamin D usual intake by sex and age group in the EsNuPI population. Differences between RS and AMS groups (n = 1448).

Group by Age	AR	AI	Boys					Girls				
			Mean	SD	Median (P5–P95)	<AR (%)	>AI (%)	Mean	SD	Median (P5–P95)	<AR (%)	>AI (%)
Calcium (mg/day), RS ^#^												
1 to <4 years	390		746 a *	184	736 (462–1064) a *	1.6		744 a	149	740 (508–997) a	0.5	
4 to <6 years	680		775 a,b *	112	772 (597–964) a,b *	20.0		743 a	161	732 (499–1026) b	37.0	
6 to <10 years	680		815 b *	192	805 (517–1148) b *	24.9		799 b *	150	791 (566–1058) c *	22.0	
Calcium (mg/day), AMS ^#^												
1 to <4 years	390		702 a *	131	697 (495–924) a *	0.5		735 a	146	727 (511–987) a	0.4	
4 to <6 years	680		835 b *	150	829 (600–1093) a *	14.9		785 b	132	780 (574–1009) b	21.5	
6 to <10 years	680		903 c *	131	897 (696–1127) b *	3.7		846 c *	171	833 (590–1147) c *	18.6	
Phosphorus (mg/day), RS ^#^												
1 to <4 years		250	943 a *	215	942 (591–1298) a *		100	939 a *	196	940 (615–1260) a *		100
4 to <6 years		440	1103 b	200	1102 (775–1434) b *		100	1068 b	175	1061 (792–1366) b		100
6 to <10 years		440	1179 c	242	1164 (809–1600) c		100	1143 c	140	1138 (920–1381) c		100
Phosphorus (mg/day), AMS ^#^												
1 to <4 years		250	826 a*	191	822 (518–1146) a *		100	870 a *	213	861 (536–1235) a *		100
4 to <6 years		440	1116 b	193	1108 (814–1446) b *		100	1042 b	160	1039 (784–1309) b		100
6 to <10 years		440	1182 c	160	1175 (932–1456) c		100	1130 c	178	1124 (846–1432) c		100
Magnesium (mg/day), RS ^#^												
1 to <3 years		170	173 a	55	170 (86–257) a		50.3	175 a	35	173 (121–235) a		54.0
3 to <6 years		230	205 b *	31	202 (159–258) b *		19.6	200 b *	33	198 (149–259) a,b *		17.9
6 to <10 years		230	220 c *	46	218 (150–306) c *		38.8	210 c *	32	208 (162–266) b *		24.5
Magnesium (mg/day), AMS ^#^												
1 to <3 years		170	178 a	47	173 (111–263) a		52.9	171	51	165 (100–265) a		45.5
3 to <6 years		230	185 a,b *	29	183 (140–236) b *		7.2	181 *	30	180 (134–231) b *		5.3
6 to <10 years		230	192 b *	27	191 (150–237) b *		7.8	185 *	45	183 (113–262) b *		16.0
Vitamin D (µg/day), RS ^#^												
1 to <4 years		15	3.20 *	2.84	2.43 (0.36–8.83) a,b *		0.6	3.05 a *	2.70	2.28 (0.34–8.55) *		0.4
4 to <6 years		15	2.77 *	2.17	2.20 (0.45–7.04) a *		0.1	3.14 b *	2.12	2.63 (0.76–7.22) *		0.1
6 to <10 years		15	2.96 *	0.96	2.85 (1.61–4.72) b *		0.0	3.09 a,b *	1.77	2.72 (0.97–6.52) *		0.0
Vitamin D (µg/day), AMS ^#^												
1 to <4 years		15	6.80 *	2.32	6.57 (3.42–10.95) *		0.4	7.51 a *	2.44	7.08 (4.30–12.10) a *		0.9
4 to <6 years		15	8.32 *	3.44	7.88 (3.51–14.63) *		4.3	7.02 a,b *	2.50	6.73 (3.46–11.55) b *		0.6
6 to <10 years		15	7.47 *	2.98	7.14 (3.18–12.86) *		1.6	6.67 b *	2.61	6.38 (2.92–11.4) b *		0.5

Average requirement (AR) and adequate intakes (AI) (EFSA, 2017). RS: reference sample (n = 707). AMS: adapted milk consumers sample (n = 741). ^#^ Variable that does not follow a normal distribution Results are expressed as the mean, standard deviation, median, and P5–P95 (in brackets). The differences between the RS and AMS sample (in same sex and age group) are indicated by asterisks (*) applying T-student and Mann–Whitney tests. Different superscript letters (a,b,c) indicate differences between age groups in the same column (same sex and same sample type: reference sample (RS) or adapted milks (AMS)),applying the Kruskal–Wallis or ANOVA tests. Different letters indicate significant differences. A *p*-Value < 0.05 was considered statistically significant.

**Table 3:** Odds ratios and 95% confidence intervals for the presence of an intake similar to or greater than the median of calcium, phosphorus, magnesium, and vitamin D, relative to family and personal factors in the reference sample (RS) of the EsNuPI children population (n = 707).

		Calcium (mg/day) (≥P50) ^†^			Phosphorus (mg/day) (≥P50) ^†^			Magnesium (mg/day) (≥P50) ^†^			Vitamin D (µg/day) (≥P50) ^†^		
Factor	Subcategories	OR	IC	*p*	OR	IC	*p*	OR	IC	*p*	OR	IC	*p*
Sex	Boys	1			1			1			1		
	Girls	0.983	0.732–1.32	0.910	0.983	0.732–1.321	0.911	1.006	0.749–1.35	0.970	0.994	0.74–1.335	0.970
Age ^¥^	1 to <4 years	1			1			1			1		
	4 to <6 years	1.075	0.716–1.612	0.728	1.042	0.695–1.564	0.842	1.000	0.672–1.488	1.000	1.042	0.695–1.564	0.842
	6-<10 years	1.031	0.740–1.436	0.857	1.000	0.718–1.393	1.000	0.993	0.678–1.455	0.973	0.987	0.708–1.375	0.937
Number of feeding bottles or glasses of milk per day	Less than 2	1			1			1			1		
	2 or more	1.681	1.218–2.320	0.002 *	1.252	0.910–1.723	0.168	1.127	0.819–1.551	0.463	1.167	0.848–1.605	0.344
Physical activity (PAL)	<P50 by sex and age	1			1			1			1		
	≥P50 by sex and age	1.023	0.761–1.374	0.880	0.892	0.664–1.199	0.449	0.873	0.650–1.173	0.366	1.269	0.944–1.705	0.114
Size of the municipality	50,001–300,000 people	1			1			1			1		
	>300,0000 people	1.277	0.950–1.716	0.106	1.25	0.930–1.68	0.140	1.336	0.994–1.797	0.055	1.449	1.077–1.949	0.014 *
Family income	≤2000 €/month	1			1			1			1		
	>2000 €/month	0.838	0.593–1.186	0.319	1.140	0.806–1.612	0.459	1.519	1.072–2.151	0.019 *	1.071	0.757–1.514	0.699
	Not known/no answer	0.877	0.607–1.267	0.485	0.903	0.625–1.305	0.587	1.133	0.784–1.638	0.505	0.860	0.595–1.243	0.422
Highest level of education achieved by one of the parents	≤10 years of education	1			1			1			1		
	Secondary education	0.297	0.108–0.819	0.019 *	0.358	0.137–0.932	0.035 *	0.810	0.343–1.915	0.632	0.402	0.161–1.005	0.051
	University studies	0.264	0.094–0.738	0.011 *	0.391	0.148–1.034	0.058	0.899	0.374–2.161	0.812	0.539	0.212–1.369	0.194
BMI for age	Normal BMI	1			1			1			1		
	Overweight and obese	0.932	0.683–1.272	0.658	1.067	0.782–1.457	0.681	0.999	0.732–1.364	0.997	1.015	0.744–1.385	0.925
	Underweight	0.408	0.190–0.879	0.022 *	0.647	0.314–1.335	0.238	0.558	0.268–1.163	0.120	0.558	0.268–1.163	0.120
Height for age	Normal height	1			1			1			1		
	High stature	1.246	0.744–2.087	0.404	1.549	0.918–2.612	0.101	1.538	0.911–2.594	0.107	1.080	0.646–1.804	0.769
	Stunting	0.836	0.519–1.349	0.463	1.006	0.625–1.619	0.981	0.854	0.53–1.378	0.518	1.472	0.909–2.385	0.116

OR: odds ratio. PAL: physical activity level. ^†^ P50 or median was calculated in the reference group for each nutrient by sex and age group, and was used to categorize children according to whether they had a usual nutrient intake below the median or superior. ^¥^ Except for magnesium (1 to <3 years, 3 to <6 years, 6 to <10 years). Underweight was defined as Z-BMI/age < −2SD, normal BMI/age was defined as Z-BMI/age −2SD to +1SD and overweight and obese as Z-BMI/age > +1SD, as per WHO guidelines [28,29]. Stunting was defined as Z-height/age < −2SD, normal BMI/age was defined as Z-height/age −2SD to +2SD and high stature as Z-height/age > +2SD, as per WHO guidelines. * A *p*-Value < 0.05 was considered statistically significant.

**Table 4:** Odds ratios and 95% confidence intervals for the presence of an intake similar to or greater than the median of calcium, phosphorus, magnesium, and vitamin D, relative to family and personal factors, in the adapted milk consumer sample (AMS) of the EsNuPI children population (n = 741).

		Calcium (mg/day) (≥P50) ^†^			Phosphorus (mg/day) (≥P50) ^†^			Magnesium (mg/day) (≥P50) ^†^			Vitamin D (µg/day) (≥P50) ^†^		
Factor	Subcategories	OR	IC	*p*	OR	IC	*p*	OR	IC	*p*	OR	IC	*p*
Sex	Boys	1			1			1			1		
	Girls	1.005	0.754–1.341	0.971	0.995	0.746–1.327	0.971	1.005	0.754–1.341	0.971	0.995	0.746–1.327	0.971
Age ^¥^	1 to <4 years	1			1			1			1		
	4 to <6 years	1.029	0.703–1.506	0.885	1.000	0.683–1.464	1.000	1.000	0.717–1.395	1.000	1.000	0.683–1.464	1.000
	6 to <10 years	1.011	0.715–1.430	0.951	1.011	0.715–1.430	0.951	0.989	0.685–1.429	0.954	1.011	0.715–1.430	0.951
Number of feeding bottles or glasses of milk per day	Less than 2	1			1			1			1		
	2 or more	2.206	1.556–3.129	0.000*	1.302	0.928–1.826	0.127	0.961	0.686–1.346	0.815	2.352	1.655–3.342	0.000 *
Physical activity (PAL)	<P50 by sex and age	1			1			1			1		
	≥P50 by sex and age	0.797	0.597–1.063	0.123	0.968	0.726–1.291	0.825	0.763	0.571–1.018	0.066	1.067	0.800–1.423	0.659
Size of the municipality	50,001–300,000 people	1			1			1			1		
	>300,000 people	0.874	0.654–1.167	0.362	1.427	1.067–1.908	0.016 *	1.441	1.078–1.927	0.014 *	0.709	0.530–0.948	0.020 *
Family income	≤2000 €/month	1			1			1			1		
	>2000 €/month	0.973	0.692–1.369	0.877	1.245	0.885–1.751	0.209	1.406	0.999–1.980	0.051	1.183	0.841–1.665	0.334
	Not known/no answer	0.854	0.598–1.219	0.385	1.128	0.790–1.61	0.507	1.489	1.042–2.129	0.029*	0.938	0.658–1.339	0.726
Highest level of education achieved by one of the parents	≤10 years of education	1			1			1			1		
	Secondary education	1.039	0.383–2.820	0.940	1.100	0.405–2.986	0.852	0.770	0.282–2.107	0.611	0.935	0.345–2.539	0.896
	University studies	0.933	0.341–2.554	0.893	0.883	0.323–2.417	0.809	0.789	0.286–2.174	0.646	1.071	0.392–2.932	0.893
BMI for age	Normal BMI	1			1			1			1		
	Overweight and obese	1.045	0.767–1.423	0.783	1.045	0.767–1.423	0.783	1.063	0.780–1.449	0.697	1.194	0.876–1.627	0.262
	Underweight	0.822	0.436–1.552	0.546	0.747	0.394–1.414	0.370	0.837	0.444–1.58	0.583	0.868	0.460–1.637	0.661
Height for age	Normal height	1			1			1			1		
	High stature	1.260	0.721–2.201	0.417	0.929	0.534–1.617	0.795	0.868	0.499–1.512	0.618	0.777	0.445–1.357	0.375
	Stunting	0.813	0.548–1.206	0.303	0.531	0.354–0.795	0.002 *	0.863	0.582–1.278	0.462	1.165	0.786–1.727	0.447

OR: odds ratio. PAL: physical activity level. ^†^ P50 or median was calculated in in the adapted milk consumer group for each nutrient by sex and age group, and was used to categorize children according to whether they had a usual nutrient intake below the median or superior. ^¥^ Except for magnesium (1 to <3 years, 3 to <6 years, 6 to <10 years). Underweight was defined as Z-BMI/age < −2SD, normal BMI/age was defined as Z-BMI/age −2SD to +1SD and overweight and obese as Z-BMI/age > +1SD, as per World Health Organization (WHO) guidelines [28,29]. Stunting was defined as Z-height/age < −2SD, normal BMI/age was defined as Z-height/age −2SD to +2SD and high stature as Z-height/age > +2SD, as per WHO guidelines. * A *p*-Value < 0.05 was considered statistically significant.

## Supplementary Materials

The following are available online at https://www.mdpi.com/2072-6643/12/6/1787/s1, Table S1: daily calcium, phosphorus, magnesium, and vitamin D usual intakes by sex and age group in the EsNuPI Study population. Results of plausible reporters. Table S2: percentage of the 18 food groups sources, in terms of the total calcium intake (in %), among Spanish Pediatric Population (EsNuPI) study by age group, in both the reference and the adapted milk consumer samples (n = 1448). Table S3: percentage of the 18 food groups sources, in terms of the total phosphorus intake (in %), among Spanish Pediatric Population (EsNuPI) study by age group, in both the reference and the adapted milk consumer samples (n = 1448). Table S4: percentage of the 18 food groups sources, in terms of the total magnesium intake (in %), among Spanish Pediatric Population (EsNuPI) study by age group, in both the reference and the adapted milk consumer samples (n = 1448). Table S5: percentage of the 18 food groups sources, in terms of the total Vitamin D intake (in %), among Spanish Pediatric Population (EsNuPI) study by age group, in both the reference and the adapted milk consumer samples (n = 1448).
