# Supplementary material for: Usual Dietary Intake, Nutritional Adequacy and Food Sources of Calcium, Phosphorus, Magnesium and Vitamin D of Spanish Children Aged One to <10 Years. Findings from the EsNuPI Study
Source: Nutrients. 2020 Jun 16;12(6):1787. doi: 10.3390/nu12061787 (PMC7353376; doi:10.3390/nu12061787)
Supplement: Supplementary file 1 [file nutrients-12-01787-s001.pdf]

Table S1. Daily calcium, phosphorus, magnesium, and vitamin D usual intakes by sex and age group in the EsNuPI Study population. Results of plausible reporters.

Group by age	AR	AI	Boys<br>Mean	SD	Median (P5–P95)	<AR (%)	>AI (%)	Girls<br>Mean	SD	Median (P5–P95)	<AR (%)	>AI (%)
Calcium (mg/day), RS #												
1–<4 years	390		713a	166	706 (454–999)a	1.6		720a	154	713 (479–984)a	0.5	
4–<6 years	680		789b	43	788 (720–861)b	20.0		716a	136	705 (511–956)b	37.0	
6–<10 years	680		834c*	195	826 (527–1169)c*	24.9		808b	136	801 (596–1044)c	22.0	
Calcium (mg/day), AMS #												
1–<4 years	390		695a	114	691 (513–889)a	0.5		717a	124	712 (523–930)a	0.4	
4–<6 years	680		845b	126	838 (648–1062)a	14.9		757b	108	756 (580–937)a	21.5	
6–<10 years	680		930c*	118	926 (744–1130)b*	3.7		849c	183	835 (574–1172)b	18.6	
Phosphorus (mg/day), RS #												
1–<4 years		250	911a*	184	910 (608–1214)a*		100	915a	195	921 (587–1228)a		100
4–<6 years		440	1131b*	124	1128 (932–1341)b*		100	1056b*	154	1048 (817–1322)b*		100
6–<10 years		440	1210c	211	1199 (882–1573)c		100	1152c	109	1149 (978–1336)c		100
Phosphorus (mg/day), AMS #												
1–<4 years		250	808a*	153	805 (560–1063)a*		100	849a	191	842 (545–1173)a		100
4–<6 years		440	1109b*	153	1104 (868–1369)b*		100	1003b*	117	1000 (815–1200)b*		100
6–<10 years		440	1220c	155	1213 (977–1487)c		100	1140c	168	1134 (874–1426)c		100
Magnesium (mg/day), RS #												
1–<3 years		170	165a	52	162 (85–254)a		44.2	173a	41	170 (111–244)a		50.3
3–<6 years		230	209b*	15	208 (186–235)b*		8.3	195b	31	192 (149–249)b*		12.6
6–<10 years		230	226c*	36	224 (171–288)c*		43.2	212c	26	210 (171–257)c*		23.3
Magnesium (mg/day), AMS #												
1–<3 years		170	176a	39	172 (120–246)a		51.7	169a	49	164 (101–255)a		43.5
3–<6 years		230	185b*	18	185 (156–217)b*		1.1	172*a	21	171 (138–208)a*		0.5
6–<10 years		230	196c*	16	195 (170–223)c*		1.9	187*b	37	186 (129–251)b*		12.9
Vitamin D (µg/day), RS #												
1–<4 years		15	2.65a*	2.46	2.62 (0.50–8.75)a.b*		0.6	2.50*	2.08	2.19 (0.41–8.03)*		0.5
4–<6 years		15	2.48b*	1.91	2.12 (0.54–6.40)a*		0.0	3.09*	2.01	2.90 (0.94–7.47)*		0.1
6–<10 years		15	2.97a,b*	1.09	2.88 (1.66–4.69)b*		0.0	3.02*	1.68	2.72 (0.92–6.68)*		0.0
Vitamin D (µg/day), AMS #												
1–<4 years		15	6.79*	2.33	6.53 (3.35–10.76)*		0.1	7.61a*	2.54	7.25 (4.55–11.01)a*		0.3
4–<6 years		15	8.04*	3.34	8.41 (3.66–15.63)*		6.4	6.74b*	2.63	6.75 (3.37–11.69)b*		0.7
6–<10 years		15	7.35*	2.96	7.33 (3.10–13.71)*		2.7	6.51b*	2.61	6.19 (2.88–10.77)b*		0.2

Average Requirement (AR) and Adequate intake (AI) (EFSA, 2017). RS: Reference Sample. AMS: Adapted milk consumers sample. #: Variable that does not follow a normal distribution. Results of plausible reporters (n = 1216) expressed as the mean, standard deviation, median, and P5–P95 (in brackets). Differences between the RS and AMS samples (in the total sample and by sex) are indicated with asterisks, applying the Chi-square and Mann–Whitney tests. The letters indicate differences between age groups for each sex and in each sample type (RS and AMS), applying the Kruskal–Wallis or ANOVA tests. Different letters indicate significant differences. P-value < 0.05 was considered statistically significant.

Table S2.- Percentage of the 18 food groups sources, in terms of the total **calcium** intake (in %), among Spanish Pediatric Population (EsNuPI) study by age group, in both the reference and the adapted milk consumer samples (n = 1448).

	Reference sample			Adapted milk consumers		
**Groups**	**1-<3 years**	**3-<6 years**	**6-<10 years**	**1-<3 years**	**3-<6 years**	**6-<10 years**
Cereals	3.6	5.7	7.4	2.7	5.8	6.3
Milk and dairy products	69.9	64.9	64.7	70.7	66.6	64.2
Eggs	1.3	1.7	1.9	1.6	1.8	1.8
Sugars and sweets	0.4	1.0	1.0	0.4	1.0	1.2
Oils and fats	0.0	0.0	0.0	0.0	0.0	0.0
Vegetables	4.7	3.8	3.3	5.0	3.3	2.6
Legumes	1.4	1.8	1.6	1.1	1.3	1.2
Fruits	2.8	2.3	2.3	3.2	2.5	2.3
Nuts	0.0	0.1	0.2	0.0	0.1	0.1
Meat and meat products	1.0	1.5	1.6	1.0	1.4	1.3
Fish and shellfish	1.1	1.3	1.5	1.1	1.1	1.3
Beverages	0.6	0.9	1.0	0.5	0.9	0.8
Sauces and condiments	0.2	0.5	0.5	0.2	0.5	0.4
Ready to cook	1.3	3.1	2.4	0.6	2.2	4.8
Appetizers	0.3	0.3	0.2	0.3	0.3	0.5
Cereal-based baby foods and supplements	2.8	0.3	0.0	3.3	0.4	0.1
Bakery and pastry	2.7	3.7	3.9	2.6	3.2	3.3
Other dairy products	5.9	7.1	6.5	5.6	7.4	7.9

Table S3.- Percentage of the 18 food groups sources, in terms of the total **phosphorus** intake (in %), among Spanish Pediatric Population (EsNuPI) study by age group, in both the reference and the adapted milk consumer samples (n = 1448).

	**Reference sample**			**Adapted milk consumers**		
**Groups**	**1-<3 years**	**3-<6 years**	**6-<10 years**	**1-<3 years**	**3-<6 years**	**6-<10 years**
Cereals	5.9	9.0	10.2	5.6	9.1	9.7
Milk and dairy products	47.6	39.2	37.3	47.1	40.4	39.0
Eggs	3.7	4.2	4.6	4.8	4.9	4.8
Sugars and sweets	2.4	4.3	5.1	2.2	4.5	5.4
Oils and fats	0.0	0.0	0.0	0.0	0.0	0.0
Vegetables	5.3	3.9	3.7	5.9	3.6	2.8
Legumes	3.4	3.6	3.3	2.8	3.0	2.6
Fruits	3.5	2.6	2.2	4.2	2.9	2.2
Nuts	0.0	0.3	0.5	0.0	0.1	0.1
Meat and meat products	10.4	13.3	13.1	10.9	12.5	11.6
Fish and shellfish	6.2	5.9	6.3	6.7	5.6	5.9
Beverages	1.1	1.1	1.4	0.7	1.1	1.1
Sauces and condiments	0.2	0.3	0.3	0.1	0.3	0.2
Ready to cook	2.4	3.1	3.0	1.2	2.9	4.6
Appetizers	0.5	0.4	0.5	0.3	0.6	0.5
Cereal-based baby foods and supplements	0.0	0.0	0.0	0.0	0.0	0.0
Bakery and pastry	3.7	4.5	4.5	3.7	4.1	4.2
Other dairy products	3.8	4.0	3.7	3.6	4.4	5.0

Table S4. Percentage of the 18 food groups sources, in terms of the total **magnesium** intake (in %), among Spanish Pediatric Population (EsNuPI) study by age group, in both the reference and the adapted milk consumer samples (n = 1448).

	Reference sample			Adapted milk consumers		
**Groups**	**1-<3 years**	**3-<6 years**	**6-<10 years**	**1-<3 years**	**3-<6 years**	**6-<10 years**
Cereals	9.7	14.9	17.4	8.1	16.1	19.3
Milk and dairy products	29.3	23.2	21.6	33.6	19.8	14.2
Eggs	1.2	1.3	1.5	1.4	1.7	1.9
Sugars and sweets	0.8	1.6	1.8	0.7	1.7	2.6
Oils and fats	0.0	0.0	0.0	0.0	0.0	0.0
Vegetables	11.9	8.8	8.7	12.5	8.4	7.6
Legumes	6.8	7.7	7.1	5.1	6.6	6.6
Fruits	16.4	12.9	10.6	17.2	14.9	12.4
Nuts	0.0	0.7	1.4	0.1	0.3	0.4
Meat and meat products	6.9	8.7	8.7	6.9	8.9	8.9
Fish and shellfish	3.9	3.9	4.4	4.0	4.0	4.8
Beverages	2.6	3.3	3.5	2.3	3.5	3.4
Sauces and condiments	0.7	1.4	1.3	0.7	1.5	1.4
Ready to cook	2.1	2.8	2.8	1.0	2.7	4.5
Appetizers	1.4	1.2	1.6	0.6	1.7	1.7
Cereal-based baby foods and supplements	0.0	0.0	0.0	0.0	0.0	0.1
Bakery and pastry	3.5	4.3	4.5	3.2	4.3	5.1
Other dairy products	2.9	3.3	3.1	2.5	3.8	5.2

Table S5. Percentage of the 18 food groups sources, in terms of the total **Vitamin D** intake (in %), among Spanish Pediatric Population (EsNuPI) study by age group, in both the reference and the adapted milk consumer samples (n = 1448).

	**Reference sample**			**Adapted milk consumers**		
**Groups**	**1-<3 years**	**3-<6 years**	**6-<10 years**	**1-<3 years**	**3-<6 years**	**6-<10 years**
Cereals	5.2	10.0	12.1	1.7	5.0	4.5
Milk and dairy products	43.5	41.0	29.6	74.4	71.4	72.2
Eggs	8.1	14.6	15.1	4.8	7.0	7.0
Sugars and sweets	0.0	0.0	0.0	0.0	0.0	0.0
Oils and fats	0.1	0.3	0.2	0.1	0.1	0.1
Vegetables	0.0	0.0	0.0	0.0	0.0	0.0
Legumes	0.0	0.0	0.0	0.0	0.0	0.0
Fruits	0.0	0.0	0.0	0.0	0.0	0.0
Nuts	0.0	0.0	0.0	0.0	0.0	0.0
Meat and meat products	0.1	0.2	0.2	0.0	0.1	0.1
Fish and shellfish	16.2	21.5	33.1	4.4	11.0	11.4
Beverages	0.0	0.1	0.0	0.0	0.1	0.0
Sauces and condiments	0.1	0.2	0.2	0.0	0.1	0.1
Ready to cook	0.8	1.8	2.3	0.3	0.4	1.2
Appetizers	0.0	0.0	0.0	0.0	0.0	0.0
Cereal-based baby foods and supplements	22.7	3.4	0.2	12.9	2.0	0.2
Bakery and pastry	1.9	4.4	4.6	0.8	1.8	1.9
Other dairy products	1.3	2.4	2.3	0.5	1.0	1.3
